# Transcription factors NF-YB involved in embryogenesis and hormones responses in *Dimocarpus Longan* Lour

**DOI:** 10.3389/fpls.2023.1255436

**Published:** 2023-09-21

**Authors:** Mengjie Tang, Xiaoli Gao, Wenyong Meng, Jindi Lin, Guanghui Zhao, Zhongxiong Lai, Yuling Lin, Yukun Chen

**Affiliations:** Institute of Horticultural Biotechnology, Fujian Agriculture and Forestry University, Fuzhou, Fujian, China

**Keywords:** *Dimocarpus longan* Lour., somatic embryogenesis, NF-YB family, nuclear transcription factor, expression profiles

## Abstract

**Introduction:**

NF-YB transcription factor is an important regulatory factor in plant embryonic development.

**Results:**

In this study, 15 longan *NF-YB* (*DlNF-YB*) family genes were systematically identified in the whole genome of longan, and a comprehensive bioinformatics analysis of *DlNF-YB* family was performed. Comparative transcriptome analysis of *DlNF-YBs* expression in different tissues, early somatic embryogenesis (SE), and under different light and temperature treatments revealed its specific expression profiles and potential biological functions in longan SE. The qRT-PCR results implied that the expression patterns of *DlNF-YBs* were different during SE and the zygotic embryo development of longan. Supplementary 2,4-D, NPA, and PP_333_ in longan EC notably inhibited the expression of *DlNF-YBs*; ABA, IAA, and GA_3_ suppressed the expressions of *DlNF-YB6* and *DlNF-YB9*, but IAA and GA_3_ induced the other *DlNF-YBs*. Subcellular localization indicated that DlNF-YB6 and DlNF-YB9 were located in the nucleus. Furthermore, verification by the modified 5'RNA Ligase Mediated Rapid Amplification of cDNA Ends (5' RLM-RACE) method demonstrated that *DlNF-YB6* was targeted by dlo-miR2118e, and dlo-miR2118e regulated longan somatic embryogenesis (SE) by targeting *DlNF-YB6*. Compared with CaMV35S- actuated *GUS* expression, *DlNF-YB6* and *DlNF-YB9* promoters significantly drove *GUS* expression. Meanwhile, promoter activities were induced to the highest by GA_3_ but suppressed by IAA. ABA induced the activities of the promoter of *DlNF-YB9*, whereas it inhibited the promoter of *DlNF-YB6*.

**Discussion:**

Hence, *DlNF-YB* might play a prominent role in longan somatic and zygotic embryo development, and it is involved in complex plant hormones signaling pathways.

## 1 Introduction

Nuclear factor-Y (NF-Y), a transcription factor ubiquitous in eukaryotes, is also known as CAATT binding factor or heme activator protein (HAP) (Edwards et al., 1998; Calvenzani et al., 2012). NF-Y was first identified in yeast and subsequently found to be widespread in animals and plants (McNabb et al., 1997); the NF-Y subunit usually has only one or two coding genes in animals and yeast, whereas it has multiple coding genes in plants. NF-Y is not only involved in plant growth development but also plays a key role in the interaction between plants and microorganisms and the environment (Petroni et al., 2012). NF-Y is composed of three distinct subunits, namely, NF-YA (or CBF-B, HAP2), NF-YB (or CBF-A, HAP3), and NF-YC (or CBF-C, HAP5) (Nakshatri et al., 1996). They interact with each other to jointly regulate the cis-acting original of CCAAT in the promoter region of downstream target genes in the nucleus, thereby activating or inhibiting the expression of downstream target genes (Baudin et al., 2015).

In *Arabidopsis thaliana*, NF-YA, NF-YB, and NF-YC have 10, 13, and 13 family members, respectively (Siefers et al., 2009). NF-YB transcription factors play an important role in plant growth development and stress resistance. At present, *NF-YB* gene family has been cloned and identified in many plants, including 13 in *Oryza sativa* L. (Zhou et al., 2018), 21 in *Zea mays* (Xu et al., 2019), and 28 in *Glycine max* (Zheng et al., 2012). The *NF-YB11* in *Triticum aestivum* responded to drought stress by regulating downstream gene expression (Zhao et al., 2022), overexpression of *NF-YB10* inhibited hypocotyl elongation and upregulated the expression of heat response genes under high temperature stress of *Arabidopsis thaliana* (Shao, 2022). The *PbNF-YB21* promoted root growth with highly lignified and enlarged xylem vessels and enhanced drought resistance by abscisic acid-mediated indoleacetic acid transport (Zhou et al., 2020). The study of Zhang et al. also showed that overexpression of *PdNF-YB21* promoted root growth and increased the biomass of poplar (Zhang Y. et al., 2021).

The NF-YB family was first reported in *A. thaliana*. *LEC1* gene was first isolated from *A. thaliana* by Lotan et al. (1998). It was found to be an essential gene in embryogenesis. The overexpression of *AtLEC1* could fully trigger embryogenetic potential and induce somatic embryos on the plant leaf surface. Then, the *LEC1* gene was cloned in corn (Zhang et al., 2002), alfalfa (Orlowska et al., 2017), cotton (Yazawa et al., 2004), and other plants. *AtNF-YB9* (*leafy cotyledon1*, *LEC1*) and *AtNF-YB6* (*LEC1-like*, *L1L*) played key roles in early embryogenesis (Lotan et al., 1998; Kwong et al., 2003). They were also important regulatory factors of plant growth development, which were involved in seed development and maturation (Tang et al., 2018). LEC1 protein interacted with gibberellin signaling inhibitor DELLA protein to regulate auxin accumulation and promote embryogenesis (Elahi et al., 2016; Hu et al., 2018; Xu J. et al., 2022), and *LEC1* could convert somatic cells into embryonic cells (Lotan et al., 1998; Orlowska et al., 2017). *NF-YB9*, *NF-YB2*, and *NF-YB3* were involved in the regulation of the flowering time of *A. thaliana* (Cai et al., 2007; Shen et al., 2020; Xu G. et al., 2022). The sequence of *L1L* was similar to that of *LEC1* with high homology (Yazawa et al., 2004). At*LEC1* and *AtL1L* could activate the CRC and SUCROSE SYNTHASE 2 (SUS2) promoters with the NF-YC subunit by interacting with the bZIP67 that binds to the ABA response elements (Yamamoto et al., 2009). Overexpression of *BnL1L* in *A. thaliana* promoted the expression of its fatty acid synthesis genes, indicating that *L1L* gene was involved in fatty acid synthesis. In rice, *OsNF-YB9* (*L1L*) interacted with SPK and played a key role in seed development (Niu et al., 2021). Paul et al. found that *L1L* gene was highly expressed in the early stage of SE of grape, and the development of grape embryos would be affected under 2,4-D treatment (Schellenbaum et al., 2008). Wei found that *L1L* gene was highly expressed in young petioles and embryogenic callus of *Liriodendron* hybrids (Wei, 2009). The cells labeled with *LhL1L* in young petioles may be residual pre-embryonic cells after seed germination, and their embryogenic properties will be lost with the maturation of petioles. *L1L* gene was involved in somatic embryogenesis by regulating the content of IAA in *Helianthus annuus × H. tuberosus* (Chiappetta et al., 2009).

Longan (*Dimocarpus longan* Lour.) belongs to the tropical and subtropical characteristic woody fruit tree; its embryo growth and development situation are closely related to fruit quality, setting rate, and yield. Therefore, it is of great significance to study the embryonic development of longan (Chen Y. et al., 2020). However, the zygote embryo of longan is wrapped in its pulp, the early embryonic development state cannot be observed, and it is difficult to obtain materials. In addition, there are genetic differences among different zygote embryos, so it is difficult to study related molecular biology in longan zygote embryos. Longan somatic embryogenesis (SE) is an excellent system for studying the embryonic development of woody plants; it has the advantages of high-frequency occurrence (Lin and Lai, 2010), high synchronization, strong regeneration ability, easy sampling, and consistent genetic background. Recently, the first whole-genome database of Sapindaceae was established by second-generation sequencing of longan (Lin et al., 2017). Then, the third-generation sequencing of the longan genome was available, which provided complete and comprehensive genomic information. The three-dimensional structure of chromatin during early SE by Hi-C technique revealed the dynamic change of genome during longan SE (Chen et al., 2023), and the improvement of longan genetic transformation system further laid the foundation for studying the molecular mechanism during longan SE (Xu et al., 2023; Zhang et al., 2023). Although the *NF-YB* family has been characterized in several plants, however, the comprehensive data regarding the evolution, expression patterns, and functions of the *NF-YB* family in longan are still unavailable. The release of longan genomic data and transcriptomics of SE provides the basis for the identification and functional analysis of longan genes. Therefore, *DlNF-YB* family were identified based on longan genomic database, their expression patterns under different exogenous hormone treatments were analyzed, and the functions of *DlNF-YB6* and *DlNF-YB9* promoter were studied, which laid a foundation for studying the regulatory mechanism of *NF-YB* family during longan SE.

## 2 Materials and methods

### 2.1 Plant materials and treatments

The synchronized embryogenic cultures at early developmental stages, including friable embryogenic callus (EC), incomplete compact pro-embryogenic cultures (ICpEC), and globular embryos (GE) were obtained by the method of Lai et al. (1997). Different development stages of zygotic embryo were collected in June to isolate the total RNA. When the young fruits emerged, cotyledon embryo stage was marked as S1; then, the zygotic embryo were collected every 5 days, marked as S2, S3, S4, S5, S6, S7, and S8, ordinal. For EC suspension culture, 2 g of 18-day subculture longan EC was transferred to MS liquid basal medium (2% sucrose) supplemented with 2,4-D (1.0 mg/L), inositol (100 mg/L) with agitation at 110 rpm at 25°C under dark conditions for 5 days (Chen et al., 2018a). Then, the suspension cell was transferred to MS liquid basal medium (2% sucrose) supplemented with 2,4-D (1.0 mg/L), KT (0.5 mg/L), and AgNO_3_ (5 mg/L) with agitation at 110 rpm at 25°C under dark conditions for 5 days. Two grams of 18-day subculture longan EC was transferred to MS liquid basal medium (2% sucrose) supplemented with 2,4-D (0.5, 1.0, 1.5, and 2.0 mg/L), IAA (0.5, 1.0, 1.5, and 2.0 mg/L), GA_3_ (3, 6, 9, and 12 mg/L), ABA (3, 6, 9, and 12 mg/L), N-1- naphthylphthalamic acid (NPA: 5, 10, 20,30, 40, and 50 mg/L), and Paclobutrazol (PP_333_: 0.05, 0.1, 0.3, 1, 2, and 3 mg/L) with agitation at 120 rpm at 25°C under dark conditions for 24 h, with three replicates. EC culture in MS liquid basal medium (2% sucrose) was used as control. All samples were frozen in liquid nitrogen immediately for 5 min after collecting and then stored at −80°C for total RNA extraction.

### 2.2 Identification and characterization of the NF-YB family in longan

The second generation of longan genome sequencing data (SRA050205) (Lin et al., 2017) and the third generation of longan genome data were provided by our research team (BioProject accession: PRJNA792504; genome sequence of *D. longan*: SRR17675476) (Chen et al., 2023). *Arabidopsis thaliana* protein sequence was downloaded from https://www.arabidopsis.org/. The latest Hidden Markov Model (HMM) for NF-YB transcription factor (PF00808) (http://pfam.xfam.org/) was used to perform HMM searches against the annotated entire protein datasets of longan with an E-value cutoff of 1e^−5^ using HMMER 3.0. The amino acid sequence of the *A. thaliana* NF-YB was used as the seed sequence. TBtools software (Chen C. et al., 2020) was used for sequence bidirectional alignment, combined with the results of NCBI analysis of the protein structure domain of the candidate sequence. The members without the domain were removed. The *DlNF-YB* family was named according to the *A. thaliana* naming method. The amino acid number (aa), isoelectric point (pI), molecular weight (MW), instability coefficient, and average hydrophilicity were predicted by Expasy Protparam (https://web.expasy.org/protparam/). The subcellular localization of DlNF-YBs were predicted by WOLF PSORT (https://wolfpsort.hgc.jp/). The gene structure of *DlNF-YB* family was analyzed by TBtools using the GFF file of ‘Honghezi ‘ longan.

### 2.3 Phylogenetic evolution and synteny analysis of DlNF-YB

*Arabidopsis thaliana* protein sequence data were downloaded from https://www.arabidopsis.org/, *Gossypium* spp. protein sequence data were downloaded from https://www.cottongen.org/, *Oryza sativa* protein sequence data were downloaded from https://rapdb.dna.affrc.go.jp/, *Litchi chinensis* Sonn (Hu et al., 2022) genome data were downloaded from https://data.mendeley.com/datasets/kggzfwpdr9/1, *Medicago sativa* L. (Du et al., 2022) genome data were downloaded from https://figshare.com/articles/dataset/Medicago_sativa_genome_and_annotation_files/12623960, *Larix kaempferi* (Li et al., 2021; Li et al., 2023) genome data were downloaded from NCBI (NCBI BioProject number: PRJNA648500), and ‘Jidanben’ longan (CRA004281) (Wang et al., 2022) genome data were downloaded from https://ngdc.cncb.ac.cn/. MEGA5.05 software was used to construct the phylogenetic tree, and the neighbor-joining method (NJ) was used to set the bootstrap value to 1,000 for repetition. The phylogenetic tree was perfected using the online tool Chiplot (https://www.chiplot.online/), and TBtools was used to predict and visualize the collinearity between longan and litchi.

### 2.4 Analysis of conserved motifs, cis-acting regulatory elements, and protein interaction analysis of DlNF-YB

Analysis of conserved motif of DlNF-YB protein sequence by MEME (http://meme-suite.org/tools/meme). The 2,000- bp sequence upstream of the transcription start site of genes in the *DlNF-YB* family was extracted from the longan genome file, and the cis-acting elements of the promoter were analyzed by PlantCARE (http://bioinformatics.psb.ugent.be/webtools/plantcare/html). The results were visualized with Tbtools. Protein–protein interactions (PPI) of DlNF-YBs were analyzed using STRING (https://string-db.org/), *A. thaliana* was selected as the model plant, and the confidence level was set to 0.400 to analyze the protein interaction of DlNF-YB family members.

### 2.5 Expression analysis of *DlNF-YB* family at the early stage of SE, different tissues, different light quality, and different temperatures

The FPKM (fragments per kilo-base of exon per million fragments mapped) values of *DlNF-YB* family members were extracted from the transcriptomes of early SE (Lin et al., 2017) (NEC [non-embryogenic callus], EC [embryogenic callus], ICpEC [incomplete compact pro-embryogenic cultures], GE [globular embryos]), different tissues (NCBI BioProject number: PRJNA326792) (young fruit, seed, flower, flower bud, leaf, pulp, root, and stem), different light qualities (Li et al., 2019) (blue, white, and dark as the control), and different temperatures (15°C, 25°C, and 35°C) (NCBI BioProject number PRJNA889670), normalized by log_2_^FPKM^. TBtoosl software was used to visualize and analyze the expression level of each member.

### 2.6 qRT-PCR analysis of *DlNF-YB* family

The total RNA of longan EC, ICpEC, GE, and EC treated with different hormones were extracted by TransZolUp kit (TransGen Biotech), and the total RNA of different development stages of the zygotic embryo was extracted by BioTeke kit (Cat. No. RP3301). The cDNA was synthesized with a Hifair^®^III 1st Strand cDNA Synthesis SuperMix for qPCR (gDNA digester plus) (Yeasen). Primer design was performed using Primer3 online software (https://primer3.ut.ee/cgi-bin/primer3/primer3web_results.cgi) (**Supplementary Table 1**). *DlACTB*, *DlEF-la*, and *DlUBQ* were used as internal control genes (Lin and Lai, 2010). The qPCR system was 20 μL, including HRbio™ qPCR SYBR^®^Green Master Mix (No Rox) (Heruibio), 8.2 μL ddH_2_O, 1 µL of 10-fold diluted cDNA, and 0.4 μL specific primer pairs, with three replicates. The reaction procedure was 95°C for 30 s, followed by 40 cycles of 95°C for 10 s and 58°C for 30 s, and 2^−ΔCT^ was used to calculate genes expression (Livak and Schmittgen, 2002; Vandesompele et al., 2002). SPSS software was used for significant analysis of gene differences, and GraphPad 8.0.2 was used for the draft.

### 2.7 Subcellular localization of DlNF-YB

The CDS sequences of *DlNF-YB6* and *DlNF-YB9* were selected to design subcellular localization primers by DNAMAN6 (**Supplementary Table 1**) and synthesized by Beijing Tsingke Biology Co., Ltd. According to Zhang X. et al., 2021, the pCAMBIA1302-GFP vector was constructed and transiently expressed in *Allium cepa* epidermal cells by *Agrobacterium*-mediated transformation. The pCAMBIA1302-GFP vector was co-transformed into *A. cepa* as a positive control. 4',6-Diamidino-2-phenylindol (DAPI) was used as a maker of nuclear localization. After 3 days of co-culture under 28°C, analysis was performed by a laser scanning confocal microscope (OLYMPUS, FV1200, Tokyo, Japan; GFP wavelength, 475 nm; DAPI wavelength, 450 nm).

### 2.8 miRNAs prediction of *DlNF-YB* family and cleavage site verification by modified 5'RLM-RACE method

The target miRNAs of *DlNF-YB* family were predicted using an online software psRNAtarget, and the expected value was set to 5. The modified 5'RLM-RACE method was used to verify the cleavage site of miRNA, and the extracted RNA of EC, ICpEC, and GE of longan was mixed with 1: 1 for reverse transcription, referenced to the FirstChoice^®^ RLM-RACE Kit instructions for the synthesis of cDNA template. The CDS sequences between 90 and 160 bp near the predicted cleavage site were selected for primer design (**Supplementary Table 1**). Nested PCR was used for amplification, the amplified products were verified by electrophoresis, and the glue was recovered and connected for transformation. After PCR verification, the bacterial liquid was tested.

### 2.9 *DlNF-YB* promoter cloning and functional analysis

The core promoter region sequences of *DlNF-YB6* and *DlNF-YB9* were selected by DNAMAN6 software to design primers (**Supplementary Table 1**); the 1,244 bp and 760 bp promoter sequences were selected to construct pCAMBIA1301-DlNF-YB6pro::*GUS* and pCAMBIA1301-DlNF-YB9pro::*GUS* fusion vector. The pCAMBIA1301::*GUS* vector containing the 35S promoter was used as a positive control. The bacteria liquid was transiently expressed in *Nicotiana benthamiana* epidermal cells by *Agrobacterium*-mediated transformation. After 3 days of co-culture, the injection leaves were sampled and placed in a refrigerator at −80°C to detect the expression level of *NbGUS*.

To examine the effects of hormones on the transcriptional level of the *DlNF-YB6* and *DlNF-YB9* promoter, 100 μmol/L IAA, ABA, and GA_3_ were sprayed on tobacco leaves 48 h after *Agrobacterium* solution injection and cultured at 25°C, 16/8 h (light/dark) for 24 h. Spraying water was used as a control to compare the expression levels of *NbGUS* in each group under different hormone treatments.

## 3 Results

### 3.1 Identification, physicochemical properties, and gene structure analysis of *DlNF-YB* family

The potential members of the *NF-YB* family were obtained by using the Hidden Markov Model of the *NF-YB* family and compared with the amino acid sequence of NF-YB family of *A. thaliana.* A total of 14 *DlNF-YB* genes were identified from the second generation of longan genome database, and 16 *DlNF-YB* genes were identified from the third generation of longan genome database. The conserved domains of the 16 candidate sequences were analyzed using SMART and Pfam databases. One sequence that did not contain CBFD_NFYB_HMF (Dlo003663) was removed. The second-generation results were correlated with the third-generation results. Finally, 15 *DlNF-YB* genes were identified, according to the similarity of *AtNF-YBs* sequences, and combined with evolutionary tree classification, the 15 genes were named as *DlNF-YB1*, *DlNF-YB2*, *DlNF-YB3*, *DlNF-YB3-like*, *DlNF-YB4*, *DlNF-YB4-like*, *DlNF-YB5*, *DlNF-YB6*, *DlNF-YB7*, *DlNF-YB7-like*, *DlNF-YB8*, *DlNF-YB9*, *DlNF-YB9-like*, *DlNF-YB10*, and *DlNF-YB11* (**Supplementary Table 2**).

The physicochemical properties of the DlNF-YBs showed that the number of aa in DlNF-YBs protein sequences ranged from 141 to 320aa. The pI ranged from 4.98 to 8.11, DlNF-YB8 and DlNF-YB10 were basic proteins, and the other 13 were acidic proteins (**Supplementary Table 2**). The hydrophilicity coefficient of DlNF-YBs were less than zero, indicating that all of them were hydrophilic proteins with different hydrophilicity gradients. The instability coefficients ranged from 32.88 to 65.85; all were unstable proteins. Subcellular localization prediction showed that DlNF-YB9-like and DlNF-YB11 were located in the cytoplasm, DlNF-YB4 and DlNF-YB6 were located in the mitochondrial matrix, and other members were located in the nucleus, indicating that DlNF-YBs may mainly play a regulatory role in the nucleus.

**Figure 1:**
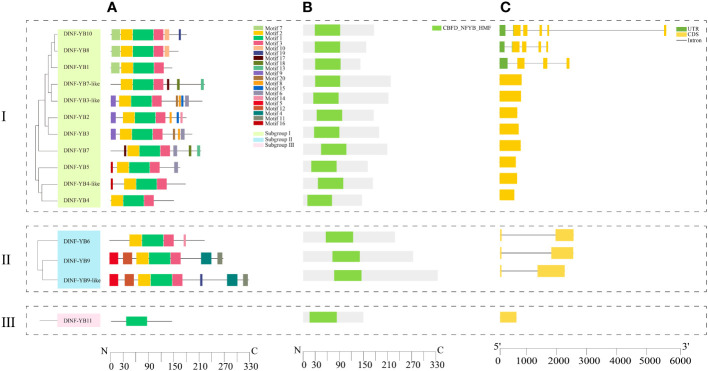
Conserved motif, domain, and gene structure of DlNF-YB family. **(A)** The motif composition of DlNF-YB proteins. The motifs, numbers 1–20, were displayed in different colored boxes; **(B)** domain of DlNF-YB proteins. Green boxes indicate CBFD_NFYB_HMF; **(C)** exon–intron structure of *DlNF-YB* genes. Yellow boxes indicate exons; green boxes indicate UTR; black lines indicate introns.

TBtools software was used to analyze and visualize the gene structure and domain of *DlNF-YB* family, and the results showed that (**Figures 1B, C**) the number of introns in each member of the *DlNF-YB* family ranges from 0 to 5, including *DlNF-YB2, DlNF-YB3, DlNF-YB3-like, DlNF-YB4, DlNF-YB4-like, DlNF-YB5, DlNF-YB7, DlNF-YB7-like*, and *DlNF-YB11* had no introns, *DlNF-YB6, DlNF-YB9* and *DlNF-YB9-like* contained 1 intron, *DlNF-YB1* contained 3 introns, *DlNF-YB8* contained 4 introns, only *DlNF-YB10* contained 5 introns. All members contain CBFD_NFYB_HMF.

### 3.2 Phylogenetic analysis and synteny analysis of *DlNF-YB*

**Figure 2:**
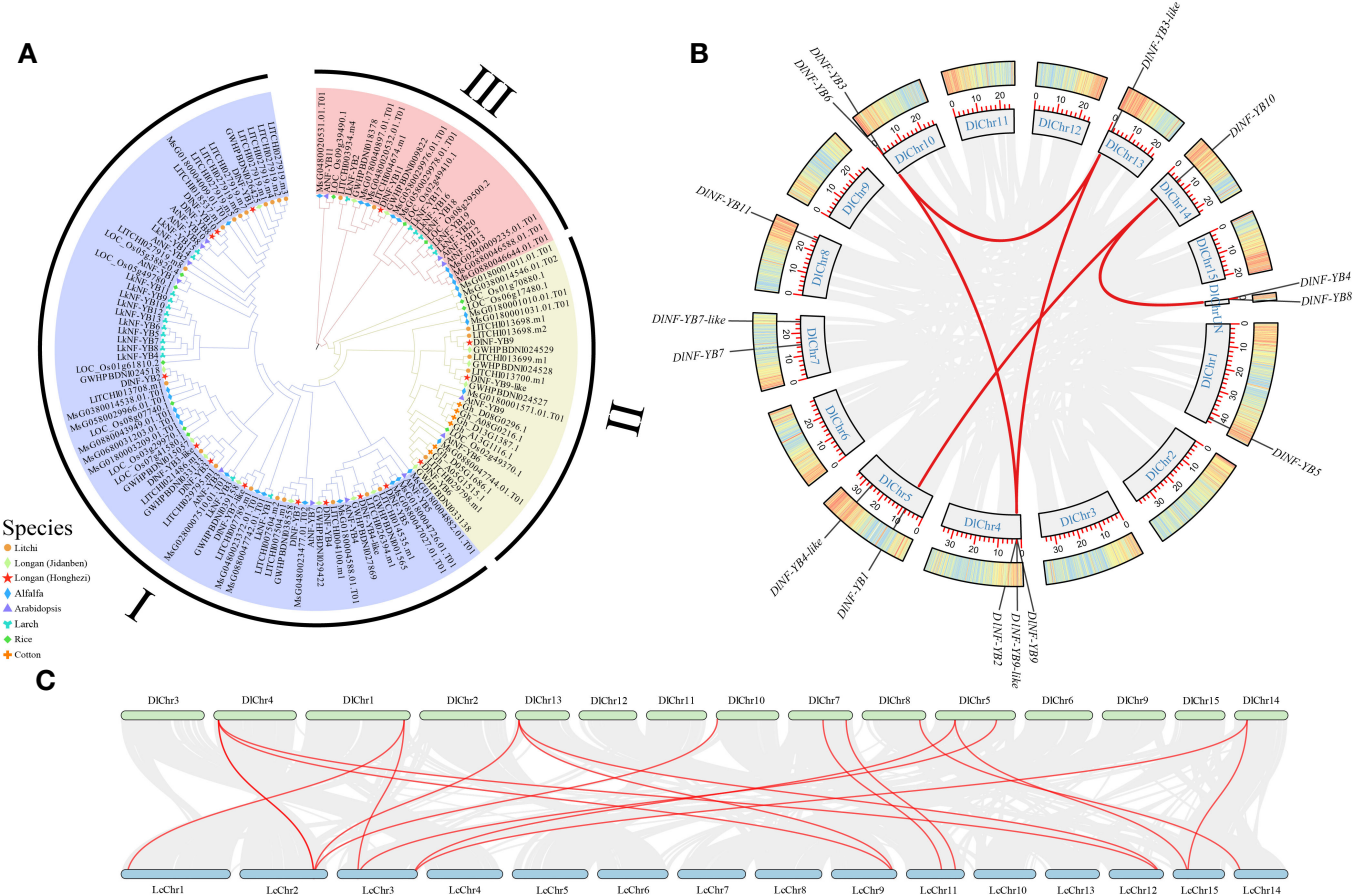
Phylogenetic tree and collinear analysis of NF-YB family. **(A)** Phylogenetic tree of NF-YB in longan, *Arabidopsis*, litchi, rice, and cotton; **(B)** chromosome localization and collinearity analysis of *NF-YB* family members in longan (gray line represents collinearity block in longan genome; red line represents linear gene pairs related to *DlNF-YB* family genes); **(C)** interspecific collinearity analysis of longan and litchi (gray line represents collinearity blocks in longan and litchi; red line represents line gene pairs related to *NF-YB* family).

In the phylogenetic tree of the NF-YB family from eight species, namely, ‘Honghezi’ longan, ‘Jidanben’ longan, *A. thaliana, Litchi chinensis* Sonn, *Gossypium* spp., *Oryza sativa*, *Medicago sativa* L., and *Larix kaempferi*, MEGA 5.05 was constructed for multiple sequence analysis according to the classification of NF-YB in *A. thaliana*, combined with the homology clustering analysis of *DlNF-YB* family. By constructing phylogenetic evolutionary tree to explore its evolutionary characteristics, the results suggested that the NF-YB could be categorized into three subgroups (**Figure 2A**). DlNF-YB6, DlNF-YB9, and DlNF-YB9-like were distributed in subgroup II; only DlNF-YB11 was distributed in subgroup III. A total of 13 members with ‘Jidanben’ longan had higher homology; DlNF-YB8 and DlNF-YB10 had high homology with litchi, indicating that different varieties of longan changed during the evolutionary process. It is speculated that there may be diversity or species-specific biological functions of DlNF-YBs due to the grouping differences and genetic relationships between species during the evolutionary process.

TBtools software was used to obtain collinearity gene pairs. The collinearity analysis of *DlNF-YB* family revealed that five tandem duplication events in seven members (**Figure 2B**); they were *DlNF-YB6* and *DlNF-YB3-like*, *DlNF-YB6* and *DlNF-YB9*, *DlNF-YB3-like* and *DlNF-YB2*, *DlNF-YB10* and *DlNF-YB1*, and *DlNF-YB9-like* and *DlNF-YB8.* The 13 *DlNF-YB* genes were unevenly distributed on 8 of the 15 chromosomes of longan, and *DlNF-YB4* and *DlNF-YB8* were located on the unknown chromosome.

To study the evolutionary relationships between longan and litchi *NF-YB* family, the interspecies collinearity analysis was conducted using their genomes. The results represented that the collinearity relationship between litchi and longan was quite close (**Figure 2C**); *NF-YB* family of litchi was also distributed on eight chromosomes, and 11 members of longan had collinearity relationship with litchi, displaying high correlation, thereby indicating that the genetic similarity between them is high in the evolutionary process.

### 3.3 Analysis of conserved motif, promoter cis-acting element, and protein interaction of *DlNF-YB*

In order to understand the distribution of protein conserved motifs of DlNF-YB family, the online software MEME was used for motif prediction analysis. As shown in **Figure 1A**, we found that motif 2 and motif 3 were only missing in DlNF-YB11; motif 1 was distributed in all members, indicating that motif 1 was highly conserved in the DlNF-YB family. In contrast, motif 17 was only detected in DlNF-YB4-like and DlNF-YB5.

**Figure 3:**
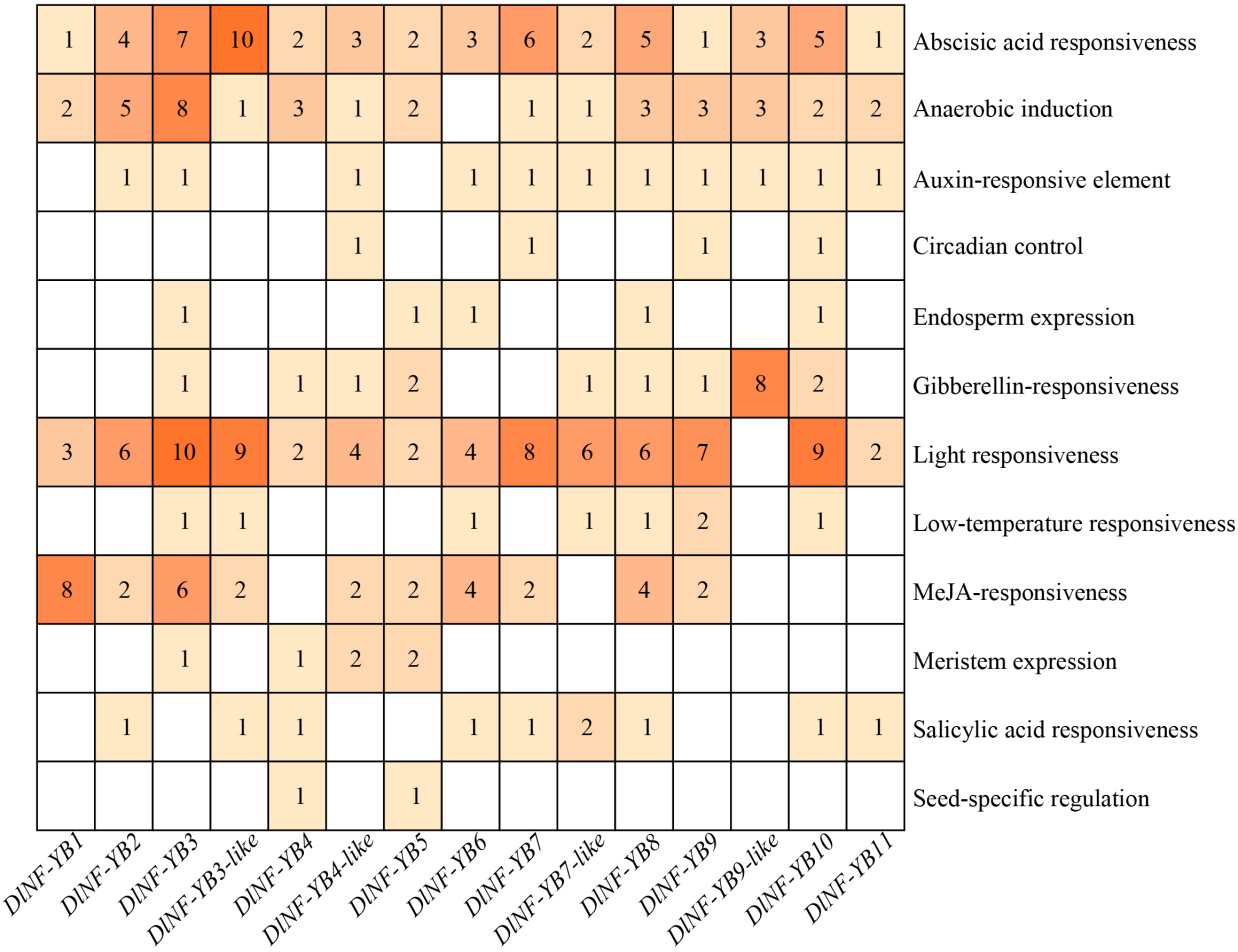
Distribution of cis-acting elements of *DlNF-YB* genes in longan. Different colors and numbers in the figure indicate the number of components, and the blank spaces indicate no components.

To further explore the functions of *DlNF-YBs* in longan, the cis-acting elements localized upstream of the translation initiation site were predicted using the PlantCARE online software. The results indicated that the *DlNF-YB* family all contains core promoter elements that ensure normal transcription and abscisic acid response elements (**Figure 3**), suggesting that they all respond to abscisic acid (ABA). In addition, there were 14 *DlNF-YBs* that could to respond to light (except *DlNF-YB9-like*), 11 *DlNF-YBs* contained auxin response elements, and 9 *DlNF-YBs* contained methyl jasmonic acid (MeJA) response elements. Seven *DlNF-YBs* (*DlNF-YB3*, *DlNF-YB6*, *DlNF-YB7-like*, *DlNF-YB8*, *DlNF-YB9*, *DlNF-YB10*, and *DlNF-YB12*) contained low temperature response elements. *DlNF-YB* family also contained a variety of elements related to plant growth and development, such as circadian control, endosperm expression elements, seed-specific development, and protein binding site.

*DlNF-YB6* and *DlNF-YB9* contained multiple core regulatory elements (**Supplementary Figure S1**). Further analysis with PlantCare found that *DlNF-YB9* contained several hormone response elements, among which only one ABRE responded to ABA, one TGA element responded to auxin, and CGTCA motif and TGACG motif responded to MeJA. Similarly, *DlNF-YB6* contained AuxRR core for auxin responsiveness, two TGACG motifs and two CGTCA motifs for MeJA responsiveness, one TCA element for salicylic acid (SA) responsiveness, and three ABRE for ABA responsiveness. It could be seen that *DlNF-YB6* and *DlNF-YB9* all responded to hormone regulation. In addition, there were also elements involved in seed-specific regulation (GCN4_motif), a large number of light-responsive elements (G-Box, GT1-motif), and low-temperature responsive elements (LTR).

**Figure 4:**
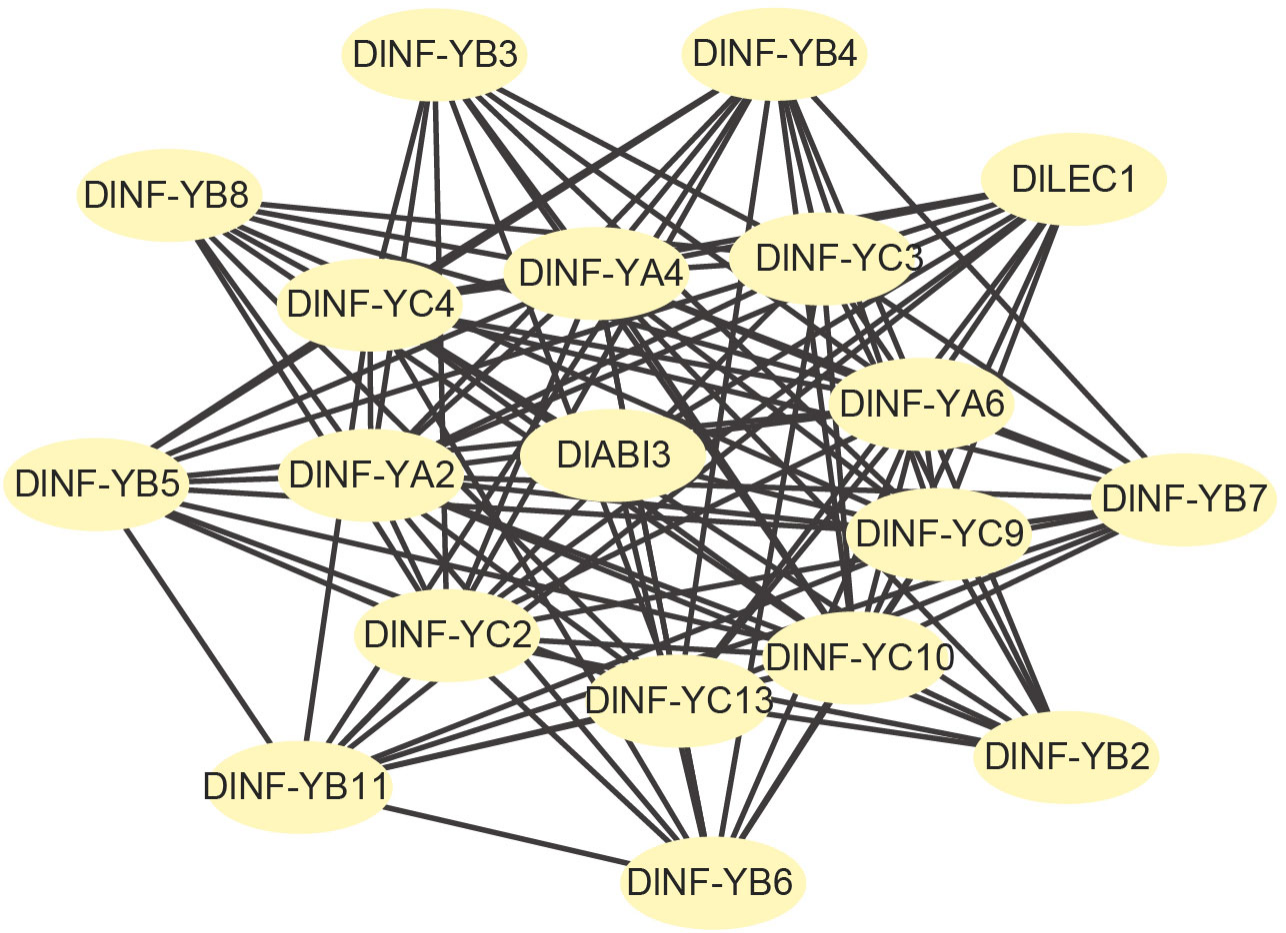
Protein–protein interactions (PPI) of DlNF-YB family members.

To better understand the interactions between DlNF-YB family and other family members. STRING software was used to analyze PPI. The results (**Figure 4**) showed that there was a strong interaction between NF-YB family members and also with subgroups A and B. DlNF-YB family not only interacts with members within the family but also interacts with ABI3.

### 3.4 Expression pattern analysis of *DlNF-YB* family

#### 3.4.1 FPKM analysis of DlNF-YB family during early SE

**Figure 5:**
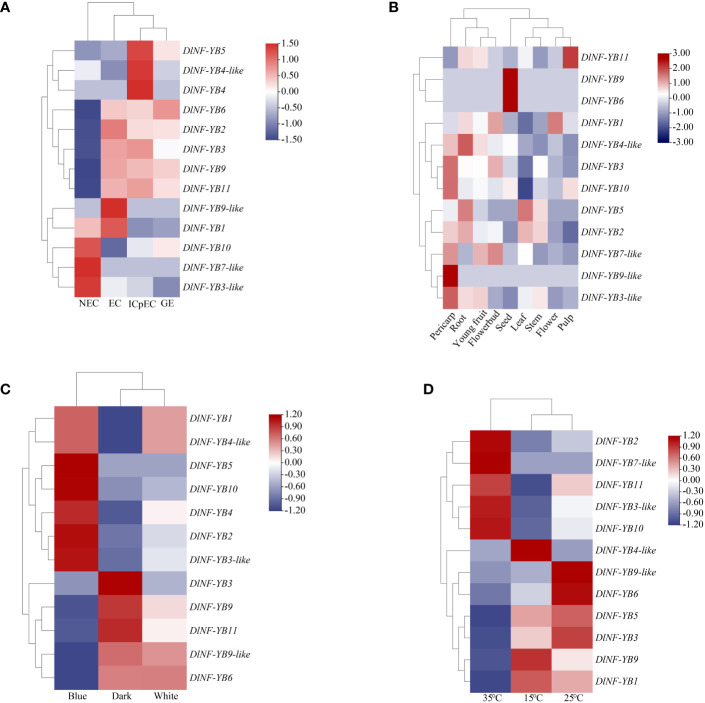
Expression patterns of *DlNF-YB* family members based on FPKM values. **(A)** FPKM value of *DlNF-YB* family during longan SE. **(B)** FPKM value of *DlNF-YB* family in different tissues. **(C)** FPKM value of *DlNF-YB* family under different light quality treatments. **(D)** FPKM value of *DlNF-YB* family under different temperature treatments. All plant materials were ‘Honghezi’ longan; different colors on the scale bar represent different transcript levels.

The FPKM values of *DlNF-YB* were analyzed based on the transcriptome of longan early SE (NEC, EC, ICpEC, and GE); *DlNF-YB2*, *DlNF-YB3*, *DlNF-YB6*, *DlNF-YB9*, and *DlNF-YB11* were mainly expressed at the early stage of SE, but barely expressed at the NEC stage (**Figure 5**). The expression level of *DlNF-YB9* was extremely high at the EC stage and gradually decreased with SE, indicating that *DlNF-YB9* played an essential role in the induction and maintenance of embryogenic callus in longan. The expression level of *DlNF-YB6* was maintained at a high level from the EC stage to the GE stage, indicating that *DlNF-YB6* had a positive regulatory effect on longan SE. *DlNF-YB3-like*, *DlNF-YB7-like*, and *DlNF-YB10* were specifically expressed at the NEC stage. *DlNF-YB7-like* was barely expressed at the early stage of longan SE. The expression level of *DlNF-YB3-like* was extremely low at the early stage of SE, and the expression level gradually decreased with SE, which was also much lower than that at the NEC stage, indicating that the function of the NF-YB family may not be limited to the early stage of SE.

#### 3.4.2 FPKM analysis of *DlNF-YB* family in different tissues

FPKM value analysis of *DlNF-YB* family based on transcriptomes of longan different tissues (young fruit, seed, flower, flower bud, leaf, pulp, root, and stem) showed that (**Figure 5**) *DlNF-YB6* and *DlNF-YB9* were highly expressed in seeds but not in other tissues, indicating that they were seed-specific genes and may play a key role in seed dormancy and embryonic development. The higher expression of *DlNF-YB5* in roots and leaves suggested that *DlNF-YB5* was involved in the growth and development of longan. *DlNF-YB1* was highly expressed in flower buds and flowers; however, it was minimal or undetected in other tissues, indicating that it was mainly involved in the development of floral organs and flowering induction. *DlNF-YB3*, *DlNF-YB3-like*, and *DlNF-YB10* were highly expressed in the pulp, indicating that the above genes may be involved in fruit development. In general, *DlNF-YB* family showed different expression patterns in different tissues, indicating that *DlNF-YB* family played an important role in the growth and development of longan.

#### 3.4.3 Analysis of FPKM values of *DlNF-YB* family under different light treatments

The FPKM values of *DlNF-YB* were analyzed based on longan EC in transcripts treated with different light qualities (white, black, and blue); the *DlNF-YB* family had different expression trends under different light quality treatments (**Figure 5**). With dark treatment compared to controls, *DlNF-YB1*, *DlNF-YB2*, *DlNF-YB3-like*, *DlNF-YB4*, *DlNF-YB4-like*, *DlNF-YB5*, and *DlNF-YB10* presented high expression in blue light treatment. It explained that blue light treatment could promote the expression of the above genes in longan EC. However, the expression levels of *DlNF-YB3*, *DlNF-YB6*, *DlNF-YB9*, *DlNF-YB9-like*, and *DlNF-YB11* under blue light treatment were lower than those under dark treatment. It can be seen that the *DlNF-YB* family also plays a critical role in light response.

#### 3.4.4 FPKM analysis of *DlNF-YB* family under different temperature treatments

FPKM value analysis of *DlNF-YB* based on the longan EC- treated transcriptome under different temperatures (15°C, 25°C, and 35°C) was performed. Compared with 25°C treatment (**Figure 5**), *DlNF-YB2*, *DlNF-YB3-like*, *DlNF-YB7-like*, *DlNF-YB10*, and *DlNF-YB11* were specifically expressed at 35°C. *DlNF-YB1*, *DlNF-YB3*, *DlNF-YB5*, and *DlNF-YB9* were significantly expressed at 15 °C; and only *DlNF-YB4-like* showed an inhibitory expression trend at 15°C and 35°C. Therefore, most of the *DlNF-YB* family may play an essential role in the self-repair process under temperature stress.

### 3.5 The qRT-PCR analysis of *DlNF-YB* family

#### 3.5.1 Expression analysis of *DlNF-YB* family during early SE

**Figure 6:**
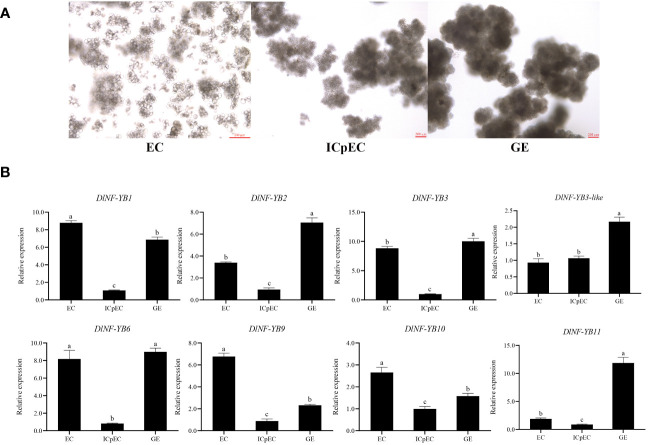
D. *longan* early stage of somatic embryogenesis and qRT-PCR analysis of longan *DlNF-YB* family during early somatic embryogenesis. Note: **(A)** Longan early stage of somatic embryogenesis. EC, embryogenic callus; ICpEC, incomplete compact pro-embryogenic cultures; GE, globular embryos. **(B)** The qRT-PCR analysis of *DlNF-YB* members the early stage of SE. The internal reference genes were *DlACTB*, *DlEF-la*, and *DlUBQ*, with three biological replicates, and significant differences with lowercase letters abc, *p* < 0.05.

Based on FPKM analysis, *DlNF-YB1*, *DlNF-YB2*, *DlNF-YB3*, *DlNF-YB3-like*, *DlNF-YB6*, *DlNF-YB9*, *DlNF-YB10*, and *DlNF-YB11* had significant differential expression during early SE of longan. The different expression patterns of these *DlNF-YBs* were analyzed by qRT-PCR. The expression trends of *DlNF-YB1*, *DlNF-YB2*, *DlNF-YB6*, *DlNF-YB9*, and *DlNF-YB11* were similar to the transcriptome data, while the qRT-PCR results of the other members were different from the RNA-seq date. It can be seen that the expression of *DlNF-YBs* in longan had a certain degree of spatial and temporal specificity during somatic embryogenesis, and the specific function needs to be further verified. The qRT-PCR result of *DlNF-YB* family at EC, ICpEC, and GE stages indicated that most of the *DlNF-YB* were highly expressed in the EC stage, except *DlNF-YB11* (**Figure 6**). The expression of *DlNF-YB1*, *DlNF-YB2*, *DlNF-YB3*, and *DlNF-YB6* at EC and GE stages were significantly higher than those at ICpEC stage. The expression of *DlNF-YB6* during longan early SE was similar to that of FPKM, which remained at a high level at the EC and GE stages, suggesting that *DlNF-YB6* had a positive regulatory effect on longan SE. *DlNF-YB9* was highly expressed at the EC stage, and *DlNF-YB11* was highly expressed at the GE stage, which showed a contradictory trend, indicating that these two genes played a key role at different stages of SE, while *DlNF-YB3-like* and *DlNF-YB10* were expressed throughout the SE stage. The above results indicated that *DlNF-YB* family was involved in longan early SE and played different roles.

#### 3.5.2 Expression analysis of *DlNF-YB* family at different development stages of zygotic embryos

**Figure 7:**
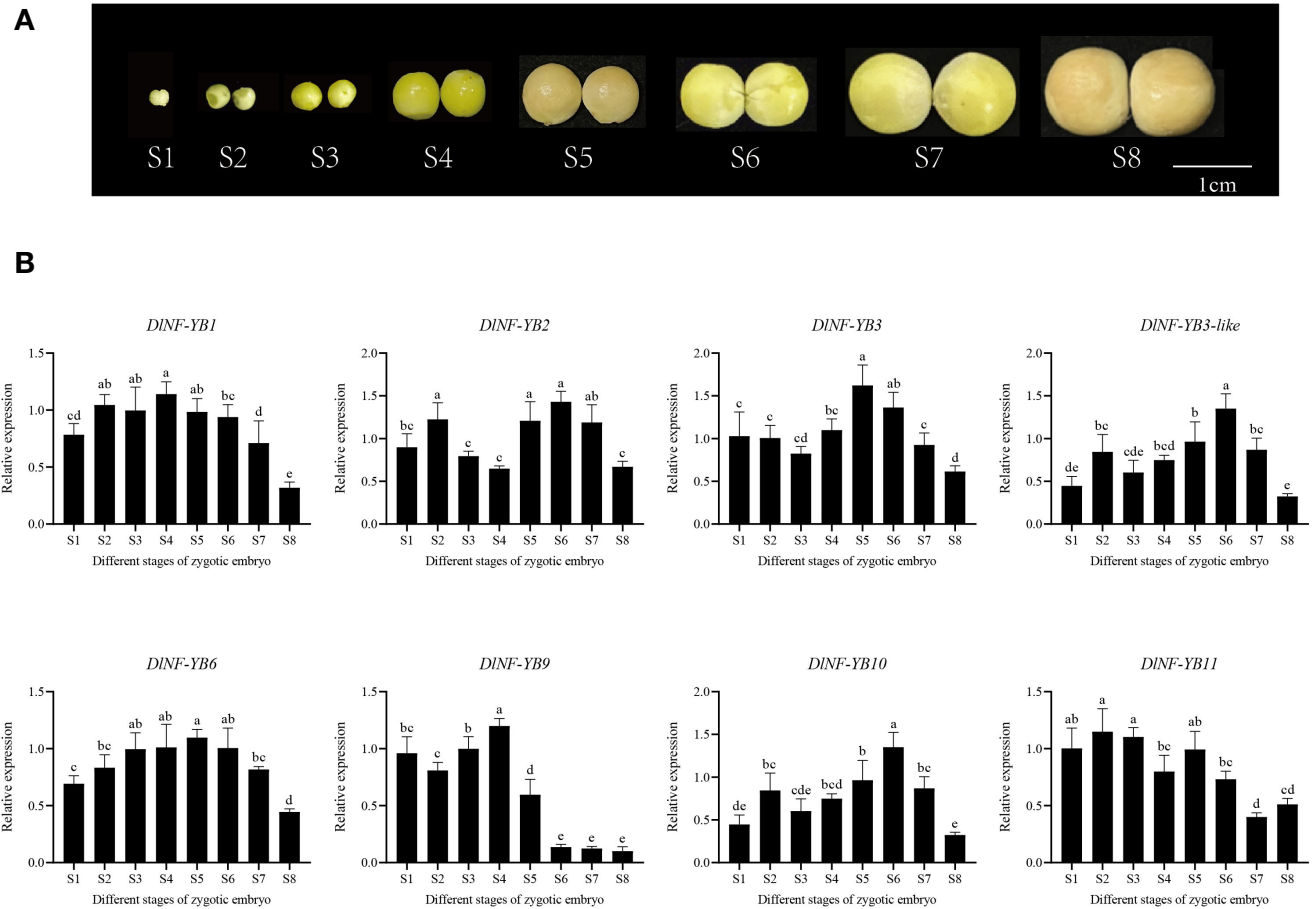
qRT-PCR analysis of longan *DlNF-YB* family at different development stages of zygotic embryos. **(A)** The different developmental stages of zygotic embryos. **(B)** The relative expression of different stages of zygotic embryos. The internal reference genes were *DlACTB*, *DlEF-la*, and *DlUBQ*, with three biological replicates, and significant differences in different lowercase letters, *p* < 0.05.

The qRT-PCR results showed that *DlNF-YBs* were detected at all development stages of zygotic embryos (**Figure 7**), and their expression patterns were different. Among them, *DlNF-YB9* was highly expressed at S1–S5 stage of zygotic embryos, and its expression was extremely low at S6–S8 stage. *DlNF-YB1* and *DlNF-YB6* showed an overall high level of expression throughout the development stage of zygotic embryos, with an initial increasing trend and a subsequent decrease, with the lowest expression at S8 stage. The expression patterns of *DlNF-YB2*, *DlNF-YB3*, *DlNF-YB3-like*, *DlNF-YB10*, and *DlNF-YB11* were similar during the development of zygotic embryos, showing a similar double-peaked “M “ trend, except that the expression of *DlNF-YB11* was the lowest at S7 stage; the others decreased to the lowest at S8 stage. Therefore, the *DlNF-YB* family may play different regulatory roles during the development of longan zygotic embryos.

#### 3.5.3 Expression analysis of *DlNF-YB* family under different exogenous hormone treatments

**Figure 8:**
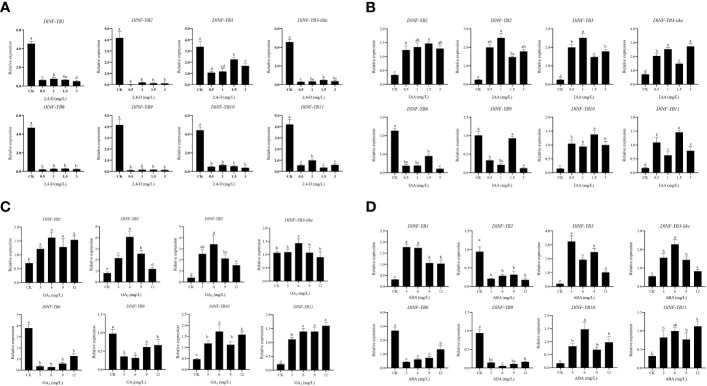
qRT-PCR analysis of *DlNF-YB* family under the 2,4-D, IAA, ABA, GA_3_ treatments. **(A)** qRT-PCR analysis of *DlNF-YB* family under the 2,4-D treatment; **(B)** qRT-PCR analysis of *DlNF-YB* family under the IAA treatment; **(C)** qRT-PCR analysis of *DlNF-YB* family under the ABA treatment; **(D)** qRT-PCR analysis of *DlNF-YB* family under the GA_3_ treatment. The internal reference genes were *DlACTB, DlEF-la*, and *DlUBQ*, with three biological replicates, and significant differences in different lowercase letters, P < 0.05.

**Figure 9:**
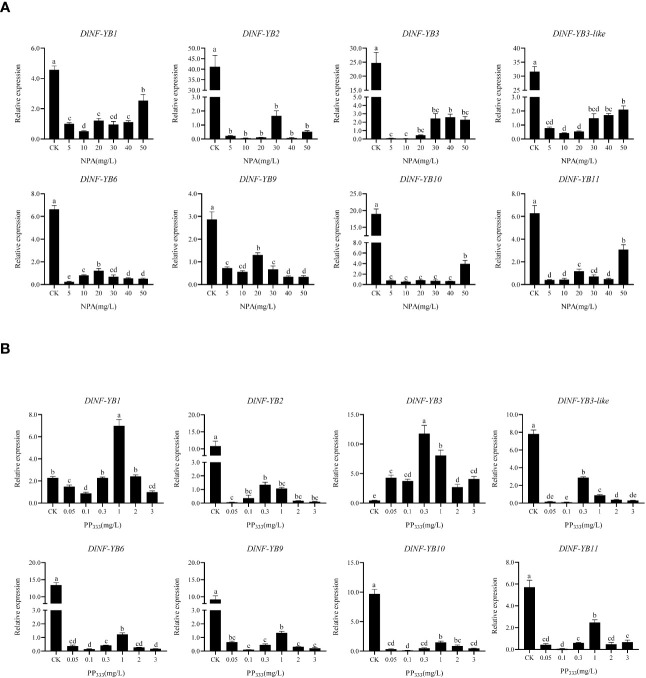
qRT-PCR analysis of *DlNF-YB* family under the NPA and PP_333_ treatment. **(A)** qRT-PCR analysis of *DlNF-YB* family under the NPA treatment; **(B)** qRT-PCR analysis of *DlNF-YB* family under the PP_333_ treatment. The internal reference genes were *DlACTB, DlEF-la*, and *DlUBQ*, with three biological replicates, and significant differences in different lowercase letters, P < 0.05.

The qRT-PCR result showed that the expression of *DlNF-YB* was significantly lower under different concentrations of 2,4-D treatment than that of the control, indicating that exogenous 2,4-D could significantly inhibit the expression of *DlNF-YB*s (**Figure 8A**). Under different concentrations of exogenous IAA treatment, the expression of *DlNF-YB1*, *DlNF-YB2*, *DlNF-YB3*, *DlNF-YB3-like*, *DlNF-YB10*, and *DlNF-YB11* were significantly higher than those of the control, indicating that exogenous IAA could promote their transcription levels. The expressions of *DlNF-YB6* and *DlNF-YB9* were significantly lower than that of the control under exogenous IAA treatment (**Figure 8B**), except that *DlNF-YB9* was not significantly different under the 1.5 mg/L IAA treatment and control. Under the treatment of 5, 10, 20, 30, 40, and 50 mg/L NPA, the transcription levels of *DlNF-YB*s were significantly lower than those of the control; especially under the treatment of 5, 10, and 20 mg/L, the expression of *DlNF-YB*s were extremely low or barely expression, indicating that exogenous NPA could significantly inhibit the expression of *DlNF-YB*s (**Figure 9A**). Meanwhile, the expression of *DlNF-YB1*, *DlNF-YB2*, *DlNF-YB3*, *DlNF-YB3-like*, *DlNF-YB10*, and *DlNF-YB11* under NPA treatment showed the opposite trend with that under exogenous IAA treatment.

The expression of *DlNF-YB* family under different concentrations of GA_3_ treatment showed that exogenous GA_3_ could significantly promote the expression of *DlNF-YB1*, *DlNF-YB2*, *DlNF-YB3*, and *DlNF-YB10*, and the expression level was the most significant under 6 mg/L GA_3_ treatment. The expression level of *DlNF-YB11* gradually increased with the increase in concentration. Among those treatment, only 6 mg/L GA_3_ can promote the expression of *DlNF-YB3-like*. However, exogenous GA_3_ significantly inhibited the transcription levels of *DlNF-YB6* and *DlNF-YB9*, which was almost the same as the expression pattern under exogenous IAA treatment (**Figure 8C**). The qRT-PCR result of *DlNF-YB* family under different concentrations of PP_333_ treatment suggested that the transcription level of *DlNF-YB1* decreased first, then increased and then decreased with the increase in PP_333_ concentration (**Figure 9B**). The expression of *DlNF-YB1* was highest under 1 mg/L PP_333_ treatment, which was significantly higher than that of the control. Treatment with different concentrations of PP_333_ significantly promoted the expression of *DlNF-YB3* but significantly inhibited the expression of *DlNF-YB2*, *DlNF-YB3-like*, *DlNF-YB6*, *DlNF-YB9*, *DlNF-YB10*, and *DlNF-YB11*.

Under different concentrations of ABA treatment, the expression levels of *DlNF-YB2*, *DlNF-YB6*, and *DlNF-YB9* were significantly lower than those of the control, indicating that exogenous ABA inhibited the transcription of *DlNF-YB2*, *DlNF-YB6*, and *DlNF-YB9* (**Figure 8D**). At the same time, the expression levels of *DlNF-YB1*, *DlNF-YB3*, *DlNF-YB3-like*, *DlNF-YB10*, and *DlNF-YB11* were significantly higher than those of the control, indicating that exogenous ABA could promote the transcription of *DlNF-YB1*, *DlNF-YB3*, *DlNF-YB3-like*, *DlNF-YB10*, and *DlNF-YB11*. In summary, *DlNF-YB* can actively respond to the treatment of exogenous hormones, and there are differences in the response mechanism to exogenous hormones. *DlNF-YB* may regulate the somatic embryo process of longan through the hormone signal transduction pathway.

### 3.6 Subcellular localization of DlNF-YB6 and DlNF-YB9

**Figure 10:**
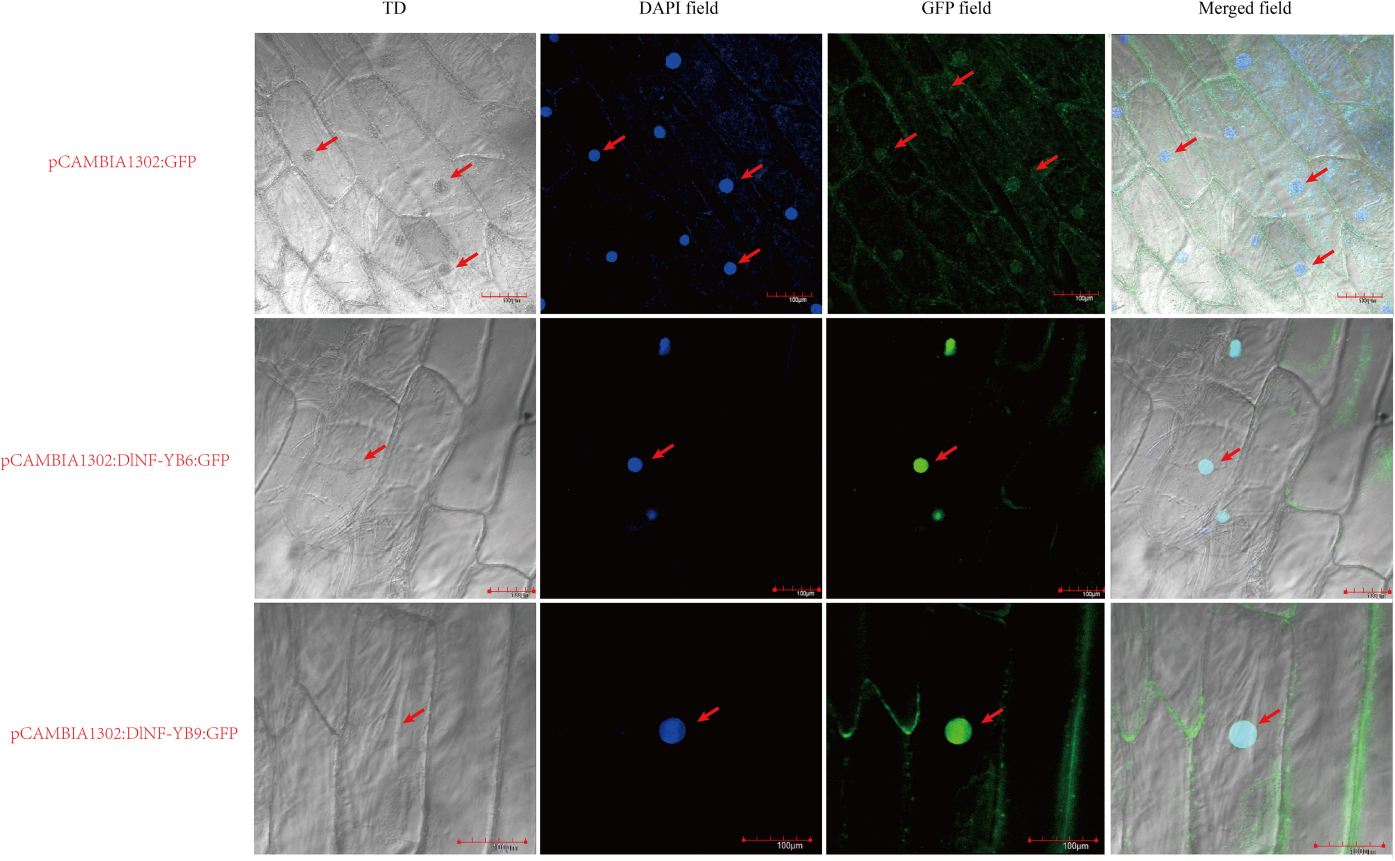
Subcellular localization of DlNF-YB6 and DlNF-YB9. TD is a transmission light channel, the scale is 100 μM, and the arrow represents the localizations of GFP and DAPI fluorescence signal in cells.

According to the prediction results of WOLF PSORT software, DlNF-YB9 (LEC1) and DlNF-YB6 (L1L) were selected for verification. The prediction results showed that DlNF-YB9 was located in the nucleus, and DlNF-YB6 was located in the mitochondrial matrix. The results showed that (**Figure 10**) all the cells of the onion inner epidermis injected with bacterial liquid had GFP fluorescence signals. The GFP fluorescence signals were distributed in the nucleus, indicating that both DlNF-YB6 and DlNF-YB9 were located in the nucleus and participated in the regulation of longan SE as nuclear transcription factors.

### 3.7 miRNAs prediction of *DlNF-YB* family and verification of cleavage site of *DlNF-YB10*

**Figure 11:**
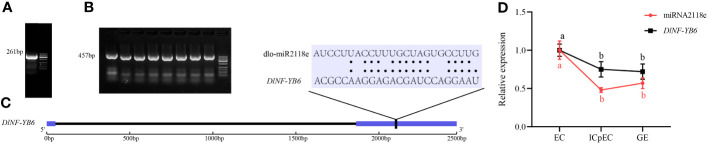
Verification of cleavage site between dlo-miR2118e and its target gene *DlNF-YB6*. **(A)** The amplified gel map. **(B)** The bacterial liquid PCR verification gel map. **(C)** The cleavage site diagram. The black line part indicates the cleavage site. **(D)** the quantitative expression of dlo-miR2118e and *DlNF-YB6* in the early stages of longan SE. U6 snRNA was used as a miRNA to quantitatively express a single internal reference gene, and *DlUBQ* was used as a target gene to quantitatively express a single internal reference gene, with three biological replicates. Different lowercase letters showed significant differences, *p* < 0.05.

The miRNA- targeted regulation of *DlNF-YB* members was predicted by psRNATarget. As shown in **Supplementary Table 3**, *DlNF-YB1*, *DlNF-YB4-like*, *DlNF-YB5*, *DlNF-YB7*, *DlNF-YB7-like*, *DlNF-YB9*, *DlNF-YB9-like*, and *DlNF-YB11* were not regulated by miRNA. The expression of *DlNF-YB2* and *DlNF-YB3-like* was regulated by dlo-miR156a in a cleavage inhibition manner, with a minimum expected value of 4. Multiple miRNAs simultaneously targeted *DlNF-YB2*, *DlNF-YB3*, and *DlNF-YB3-like*. Based on different expectations, the cleavage site of the *DlNF-YB6* (*L1L*) target gene was verified by the improved RLM-RACE method. The results indicated that *DlNF-YB6* was targeted by dlo-miR2118e, where the cleavage site was the classic 10th CCU/UUG (**Figure 11**). The expression analysis of miRNA and its target genes in the three stages of longan SE showed that both of them were downregulated in the early stage of longan SE from EC to ICpEC, while at the stage of ICpEC to GE, dlo-miR2118e was upregulated and its target gene *DlNF-YB6* was downregulated, indicating that there was a negative regulatory relationship between the ICpEC to GE. In conclusion, dlo-miR2118e could regulate longan SE by targeting *DlNF-YB6*.

### 3.8 Functional verification of *DlNF-YB6* and *DlNF-YB9* promoter

**Figure 12:**
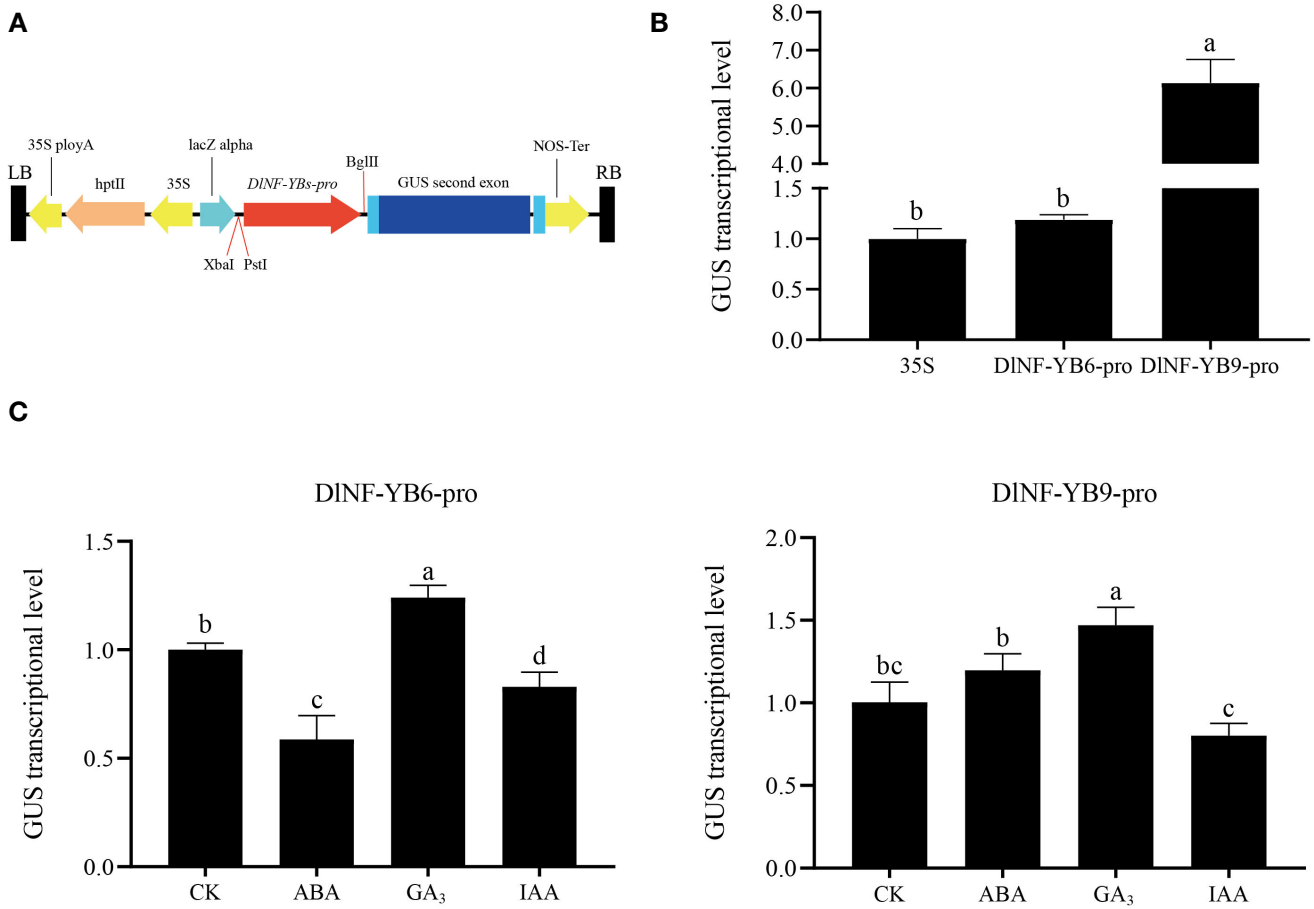
*GUS* transcriptional level generated by *35S* promoter and *DlNF-YB6/9* promoter in *N. benthamiana* and *GUS* transcriptional level generated by *35S* promoter and *DlNF-YB6/9* promoter in *N. benthamiana* under different hormone treatments. **(A)** The schematic diagram of *DlNF-YB6/9-pro* vector fragment; **(B)** the expression level of transiently transformed tobacco *GUS* gene; **(C)** the expression level of *GUS* gene in transformed tobacco leaves under different hormone treatments. *Nb18S* was used as an internal reference gene for *N. benthamiana GUS* gene expression, with three biological replicates, and significant differences with lowercase letters abc, *p* < 0.05.

The promoter of *DlNF-YB6* and *DlNF-YB9*, which contained transcription start sites and important cis-acting element regions, were cloned from longan gDNA for pCAMBIA1301-DlNF-YB6pro::*GUS* and pCAMBIA1301-DlNF-YB9pro::*GUS* vector construction. The transient transformation in *N. benthamiana* showed that the promoters of *DlNF-YB6* and *DlNF-YB9* could promote the expression of *GUS*, among which *DlNF-YB9* had the strongest ability to actuate the expression of *GUS*, and the activation of *DlNF-YB9* promoter was approximately 6.14 times that of *35S* promoter (**Figure 12**). The expression of *GUS* gene under different hormone treatments was compared by using *N. benthamiana* leaves infected with pCAMBIA1301:*GUS* bacterial solution and sprayed with water as control. The highest *GUS* transcription level was detected in the GA_3_-treated samples, and the *DlNF-YB6* and *DlNF-YB9* promoter- driven *GUS* transcription levels under GA_3_ treatment were approximately 1.5-fold compared with the control.

Under ABA treatment, *DlNF-YB6* promoter-driven *GUS* gene was inhibited, which was approximately 50% of the control; on the contrary, the *DlNF-YB9* promoter-driven *GUS* gene expression, which was approximately 1.2 times of the control. Meanwhile, IAA-treated samples presented a repressed effect, both of which were approximately 80% –83% of the control.

## 4 Discussion

### 4.1 *DlNF-YB* may be involved in longan somatic and zygotic embryogenesis

*AtNF-YB9* (*LEC1*) and *AtNF-YB6* (*L1L*) play important roles in early embryogenesis (Lotan et al., 1998; Kwong et al., 2003). In our study, based on the genome-wide identification of the *DlNF-YB* family and its expression analysis during early SE and zygotic embryo, the results indicated that a large number of longan *NF-YB* genes were highly expressed at EC to GE stage and the development of the zygotic embryo. *DlNF-YB6* and *DlNF-YB9* were expressed specifically at EC stage and changed with the process of SE, suggesting that *DlNF-YB6* and *DlNF-YB9* were specific to the early stage of longan SE and played a crucial role in the development stage of EC. Chen et al. (2018b) found that ectopic expression of *GhNF-YB22* promoted the formation of EC and promoted the embryonic development of cotton. RNA-seq shows the change trend of gene expression in the whole sample, but it cannot guarantee that the change trend of every gene is consistent with qRT-PCR (Everaert et al., 2017). In our study, the qRT-PCR trends of some members were different from RNA-seq date, presumably due to samples not being from the same batch and related to primer selection. The cleavage site of *DlNF-YB6* was verified by the modified RLM- RACE method, and the result suggested that *DlNF-YB6* was targeted by dlo-miR2118e. Meanwhile, both showed a negative regulatory relationship from ICpEC to GE. These results clearly show that *DlNF-YBs* played a prominent role in longan SE and zygotic embryo development. Niu et al. (2021) found that *OsNF-YB7* and *OsNF-YB9* played a crucial role in the formation of rice seeds, and the absence of *OsNF-YB7* and *OsNF-YB9* would lead to abnormal seed development and death. According to the FPKM values of different tissues of longan, *DlNF-YB6* and *DlNF-YB9* were highly expressed in the seed, suggesting that they might be involved in longan seed development. *AtNF-YB2* played a key role in the flowering processes (Cai et al., 2007), and *Arabidopsis* HAP3b controlled flowering time by regulating the flowering gene *AtFT* (Kumimoto et al., 2008). Furthermore, *DlNF-YB1* was highly expressed in flower buds and flower stages, indicating that it played an important role in the flowering process of longan.

### 4.2 *DlNF-YB* involved in longan SE through hormones and stress response

Hormones are involved in the growth and development of most plants. Gao (2001) found that low concentration of ABA could promote SE and further regulated SE in carrot. Moreover, ABA can also improve the tolerance of many plant embryos and inhibit premature germination of embryos, thereby improving the quality of SE (Vahdati et al., 2008; Rai et al., 2011). The expression of *AsNF-YB3* was promoted by exogenous ABA during tobacco seed germination (Sun et al., 2016). In *Jatropha curcas* seedlings, *JcNF-YB3* and *JcNF-YB10* genes were upregulated to varying degrees under the action of exogenous ABA (Huang et al., 2017). From our study, exogenous ABA could significantly upregulate the expression of most members of the *DlNF-YB* family, but inhibit *DlNF-YB2*, *DlNF-YB6*, and *DlNF-YB9* expression. Therefore, *DlNF-YB* might be involved in ABA signal transduction and also participate in longan SE. It was found that plants treated with NPA had a phenotype similar to PIN family protein mutants, and NPA may play a role by inhibiting *PIN* family (Abas et al., 2021). Subsequently, Yang et al. (2022) found that IAA and NPA had certain similarities in binding mode, but NPA molecules were larger and bound to PIN1 in a higher affinity way, which verified the efficient inhibition of NPA. Meanwhile, NPA could directly target PIN proteins. Supplementary 2,4-D, NPA in longan EC notably inhibited the expression of *DlNF-YBs*. IAA suppressed the expression of *DlNF-YB6* and *DlNF-YB9*, but the other *DlNF-YBs* were induced by IAA. Moreover, under the addition of NPA, the expression trends of *DlNF-YB1*, *DlNF-YB2*, *DlNF-YB3*, *DlNF-YBlike*, *DlNF-YB10*, and *DlNF-YB11* showed an opposite trend compared with that under IAA treatment. It is speculated that *DlNF-YB* family may participate in the process of NPA inhibiting the effect of *PIN* family. Meanwhile, GA_3_ inhibited the expression of *DlNF-YB6* and *DlNF-YB9*, while it promoted the expression of other members. Promoters of *DlNF-YB6* and *DlNF-YB9* driven *GUS* gene expression were different. The results of spraying exogenous hormones showed that GA_3_ promoted the expression of *GUS* gene driven by *DlNF-YB6* and *DlNF-YB9* promoter, while IAA inhibited it, consistent with the study that GA_3_ significantly induced *DlRan3A* promoter activity (Tian et al., 2015). GA_3_ is involved in somatic embryogenesis and regulates the expression of transcription factors related to somatic embryogenesis. By supplementing exogenous GA_3_ to change endogenous GA levels, it is also related to the acceleration of starch hydrolysis by GA_3_ by increasing α-amylase activity (Lai and Chen, 2002; Rudus et al., 2002). Therefore, *DlNF-YBs* may also play a role in the energy metabolism signal transduction pathway during longan SE. However, ABA induced the activities of *DlNF-YB9* promoter, whereas it inhibited the *DlNF-YB6* promoter activities. ABA plays an essential role in promoting the maturation of plant somatic embryos; meanwhile, the content of ABA was different during longan SE (Lai and Chen, 2002; Langhansová et al., 2004). It was found that *DlNF-YB6* and *DlNF-YB9* played a role in different stages of longan SE. Although the opposite effects of ABA on transcription of *DlNF-YB6* and *DlNF-YB9* promoters suggest that they may be involved in ABA signaling pathways, their main functions and action time were different during longan SE. The result now proves that the *DlNF-YB* family was involved in various hormone signal transduction pathways and affected longan SE.

Previous studies have shown that for longan in the embryonic development period, if the temperature continues at 34°C–38°C, it will lead to embryonic development stagnation and the formation of small fruit (Nong et al., 2006). In addition, high temperature stress was carried out during the proliferation of longan EC, and it was found that the proliferation rate of longan EC increased at 35°C, but the growth of longan EC stagnated at 40°C (Wang, 2019). The expression of *DlERF6* increased at 35°C in the early three stages of longan embryo development. Overexpression of *DlERF6* increased TAG (triacylglycerol) content at high temperature and inhibited longan SE (Zhang, 2022). The expression of *DlNF-YB* family under different temperature treatments showed that, except for *DlNF-YB2* and *DlNF-YB10*, the expression of others were promoted at high temperature (35°C) or low temperature (15°C). In *A. thaliana*, *AtNF-YB3* forms NF-Y complex with *AtNF-YA2* and *AtNF-YC10* (Sato et al., 2014), which was involved in plant heat stress response and improved plant survival rate, and overexpression of *AtNF-YB3* plants enhanced heat resistance (Sato et al., 2019). *AtNF-YB10* inhibited hypocotyl elongation induced by mild and heat stresses under light, suggesting that *AtNF-YB10* may be a negative regulator of thermomorphogenesis (Shao, 2022). Analysis of the cis-acting elements of the promoter revealed that the *DlNF-YB* family contained many CCAAT boxes. The study found that this response element could confer plant resistance to cold stress (Shi and Chan, 2014). In *Phaseolus vulgaris*, miR2118 was upregulated under ABA and drought stress (Valdés-López et al., 2010). In summary, the *DlNF-YB* family can participate in the process of longan SE by responding to abiotic stress.

## 5 Conclusions

Based on the genome data of *D. longan*, we conducted an in-depth analysis of the *DlNF-YB* family, and 15 *DlNF-YB* genes were identified in the *D. longan* genome. A comprehensive bioinformatics analysis of *DlNF-YB* family was performed, under different light and temperature treatments, revealing its specific expression profiles and potential biological functions in longan SE. In addition, *DlNF-YB6* was targeted by dlo-miR2118e, and dlo-miR2118e could regulate longan SE by targeting *DlNF-YB6.* Through the study of promoters, *DlNF-YB6* and *DlNF-YB9* promoters could generate the expression of *GUS* in different degrees. Gene expression analysis showed that *DlNF-YBs* were expressed in different degrees during longan SE, suggesting their specific functions, and *DlNF-YBs* were regulated by ABA, GA_3_, and IAA, revealing the *DlNF-YBs* response to hormone signal pathways. This study explained that *DlNF-YBs* play a prominent role during longan SE, which provided a reference for better research on the functions of *DlNF-YBs*.

## Data availability statement

The datasets presented in this study can be found in online repositories. The names of the repository/repositories and accession number(s) can be found in the article/**Supplementary Material**.

## Author contributions

MT: Data curation, Formal Analysis, Writing – original draft. XG: Writing – original draft, Investigation, Methodology, Project administration. WM: Resources, Writing – original draft, Methodology. JL: Data curation, Methodology, Writing – original draft. GZ: Project administration, Software, Writing – original draft. ZL: Writing – review & editing, Funding acquisition. YL: Writing – review & editing, Funding acquisition. YC: Funding acquisition, Writing – review & editing.

## Supplementary material

Supplementary Figure 1 The nucleotide sequence of *DlNF-YB6* and *DlNF-YB9* promoter. Note: The putative regulatory elements and hormone-responsive elements on both strands are shown in bold and underlined, which was performed by the PlantCARE database.
