# Supplementary material for: Transcription factors NF-YB involved in embryogenesis and hormones responses in Dimocarpus Longan Lour
Source: Front Plant Sci. 2023 Sep 21;14:1255436. doi: 10.3389/fpls.2023.1255436 (PMC10570845; doi:10.3389/fpls.2023.1255436)
Supplement: Supplementary file 1 [file Table_1.docx]

**Supplementary Material**

**Supplementary Table 1** Primers Used in Cloning, qRT-PCR, and Vector Constructions

| Primer Name | Primer sequence（5’ to 3’） | Function |
| --- | --- | --- |
| *DlNF-YB6-pro-F* | AACTGCAGACAATGTGTATATATAGAGTTTG | Construction of *DlNF-YB-pro*:GUS |
| *DlNF-YB6-pro-R* | GAAGATCTCTCTCTCTGTGGCAAGTCTCTATC |  |
| *DlNF-YB9-pro-*F | GCTCTAGAAAATCCGAACTAAAAAATGTCCACCCCTA |  |
| *DlNF-YB9-pro-*R | GAAGATCTATTGTCTCTTTAGTGGGTGCTGTCA |  |
| *NbGUS-qF* | CGACTGGGCAGATGAACAT | qRT-PCR for promoter functional analysis |
| *NbGUS-qR* | ATACTCCACATCACCACGCT |  |
| *Nb18S- qF* | CCTGAGAAACGGCTACCACAT | Reference gene for qRT-PCR  in *N. benthamiana* |
| *Nb18S- qR* | CACCAGACTTGCCCTCCA |  |
| *DlNF-YB6-1302-F* | TGCCATGGATGGATTGCGGAGGCTTT | Construction of *DlNF-YB*:GFP |
| *DlNF-YB6-1302-R* | GGACTAGTTTTGTCATGAGCATAACC |  |
| *DlNF-YB9-1302-*F | TGCCATGGATGGAAGGAGGATACAG |  |
| *DlNF-YB9-1302-*R | GGACTAGTTTTATAGTGTGCATACGG |  |
| 5' RACE outer primer | GCTGATGGCGATGAATGAACACTG | RLM-RACE (Nested PCR Round 1) |
| 5' RACE inner primer | CGCGGATCCGAACACTGCGTTTGCTGGCTTTGATG | RLM-RACE (Nested PCR Round 2) |
| *DlNF-YB6-*outer | TTGTTAGCATTACCATCATTGAAGTA | RLM-RACE (Nested PCR Round 1) |
| *DlNF-YB6-*inner | ACTCAACTGCTGGCCTCTTCACTAG | RLM-RACE (Nested PCR Round 2) |
| *DlNF-YB6-q*F | ATGAATGCACGGTGAGGGA | qRT-PCR for RLM-RACE analysis |
| *DlNF-YB6-q*R | CGTTATGAAGCTGATGTACTCTGAGA |  |
| dlo-miR2118e | GTTCCGTGATCGTTTCCATTCCTA |  |
| U6 snRNA | CGATACAGAGAAGATTAGCATGG | qRT-PCR for RLM-RACE analysis (Reference gene) |
| *DlNF-YB1-*qF | TCTCCGAGTTCATCAGCTTCA | qRT-PCR |
| *DlNF-YB1-*qR | CCTCAAATCCAAGTGTTGCCA |  |
| *DlNF-YB2-*qF | AGGAGTATGTGGACCCTTTGA |  |
| *DlNF-YB2-*qR | ACCACCACCACCATGATGAT |  |
| *DlNF-YB3*-qF | AGTGTTGTTGTTGGAGGACG |  |
| *DlNF-YB3*-qR | ACCACCATACATGCCACCAT |  |
| *DlNF-YB3-like*-qF | TCTGGCGCGTATCATCATCA |  |
| *DlNF-YB3-like*-qR | ACCTTGACCTAACAGCTCCA |  |
| *DlNF-YB6*-qF | CACGGTGAGGGAACAAGA |  |
| *DlNF-YB6*-qR | TTGGCGTCGTCTGAGATT |  |
| *DlNF-YB9-*qF | AACGCAAGACCATCACCTC |  |
| *DlNF-YB9-*qR | AACCTTACCACAATCACCG |  |
| *DlNF-YB10-qF* | TCTCCGAGTTCATCAGCTTCA |  |
| *DlNF-YB10-qR* | CTCAAACCCTAGAGTTGCCA |  |
| *DlNF-YB11-*qF | TCATGCCCATTCAAGAAACGA |  |
| *DlNF-YB11-*qR | TGCGGGAATGTGAGGAGTTA |  |
| *DlUBQ-qF* | GCCGACTACAACATCCAGAAG | qRT-PCR (Reference gene) |
| *DlUBQ-qR* | GCTTGGTGTAGGTCTTCTTCTT |  |
| *DlEF1α-qF* | GATAATTCCCACCAAGCCCAT |  |
| *DlEF1α-qR* | GGGTCCTTCTTCTCAACACTCT |  |
| *DlACTB-qF* | TGCTATCCTTCGGTTGGACC |  |
| *DlACTB-qR* | CGGACGATTTCCCGTTCAG |  |

**Supplementary Table 2** Physicochemical properties of longan DlNF-YB family

| Gene ID | Gene name | Location | Number of  amino acid | pI | Molecule weight/kD | Instability index | Grand average  of hydropathicity | Subcellular localization |
| --- | --- | --- | --- | --- | --- | --- | --- | --- |
| Dlo003203 | *DlNF-YB5* | Chr1:42638579:42639076:(-) | 165 | 6.06 | 18.57 | 47.38 | -1.096 | Nuclear |
| Dlo008294 | *DlNF-YB9* | Chr4:1881509:1883967:(-) | 261 | 5.27 | 28.86 | 52.42 | -0.607 | Nuclear |
| Dlo008295 | *DlNF-YB9-like* | Chr4:1893327:1895499:(-) | 320 | 4.98 | 34.56 | 48.49 | -0.505 | Cytoplasm |
| Dlo008303 | *DlNF-YB2* | Chr4:1949817:1950359:(-) | 180 | 6.08 | 19.19 | 32.88 | -0.661 | Nuclear |
| Dlo011000 | *DlNF-YB1* | Chr5:8415778:8417915:(+) | 146 | 6.14 | 15.72 | 49.12 | -0.711 | Nuclear |
| Dlo012165 | *DlNF-YB4-like* | Chr5:26429177:26429713:(-) | 178 | 6.44 | 19.85 | 37.77 | -0.849 | Nuclear |
| Dlo016488 | *DlNF-YB7* | Chr7:14968930:14969577:(-) | 215 | 6.34 | 24.39 | 57.50 | -1.021 | Nuclear |
| Dlo016984 | *DlNF-YB7-like* | Chr7:25060966:25061640:(+) | 224 | 6.23 | 24.69 | 42.75 | -0.708 | Nuclear |
| Dlo018959 | *DlNF-YB11* | Chr8:25010729:25011154:(-) | 141 | 6.33 | 16.10 | 65.85 | -0.726 | Cytoplasm |
| Dlo021117 | *DlNF-YB6* | Chr10:328223:330690:(-) | 218 | 5.92 | 24.27 | 38.02 | -0.686 | Mitochondrial matrix |
| Dlo021120 | *DlNF-YB3* | Chr10:344624:345208:(-) | 194 | 6.30 | 20.68 | 38.69 | -0.701 | Nuclear |
| Dlo026931 | *DlNF-YB3-like* | Chr13:1326375:1327031:(-) | 218 | 6.66 | 22.07 | 45.99 | -0.673 | Nuclear |
| Dlo029371 | *DlNF-YB10* | Chr14:5315007:5320100:(+) | 181 | 8.38 | 19.52 | 45.29 | -0.733 | Nuclear |
| Dlo033721 | *DlNF-YB8* | unanchor145_1_200317:157904:159389:(-) | 161 | 8.81 | 17.61 | 46.17 | -0.584 | Nuclear |
| Dlo034761 | *DlNF-YB4* | unanchor298_1_25000:11536:11988:(-) | 150 | 5.29 | 16.90 | 41.20 | -0.867 | Mitochondrial matrix |

**Supplementary Table 3** Prediction of miRNAs of *DlNF-YB* family members

| Target | miRNA | Expectation | Inhibition |
| --- | --- | --- | --- |
| *DlNF-YB2* | dlo-miR156a | 4.5 | Cleavage |
|  | dlo-miR159b | 3 | Cleavage |
| *DlNF-YB3* | dlo-miR534a | 4.5 | Cleavage |
|  | dlo-miR401 | 5 | Cleavage |
|  | dlo-miR417 | 5 | Cleavage |
| *DlNF-YB3-like* | dlo-miR156a | 4 | Cleavage |
|  | dlo-miR447a | 4.5 | Cleavage |
| *DlNF-YB4* | dlo-miR440 | 5 | Translation |
| *DlNF-YB6* | dlo-miR2118e | 5 | Cleavage |
| *DlNF-YB8* | dlo-miR172a | 3.5 | Cleavage |
