# Supplementary figures and images for: Transcription factors NF-YB involved in embryogenesis and hormones responses in Dimocarpus Longan Lour
Source: Front Plant Sci. 2023 Sep 21;14:1255436. doi: 10.3389/fpls.2023.1255436 (PMC10570845; doi:10.3389/fpls.2023.1255436)

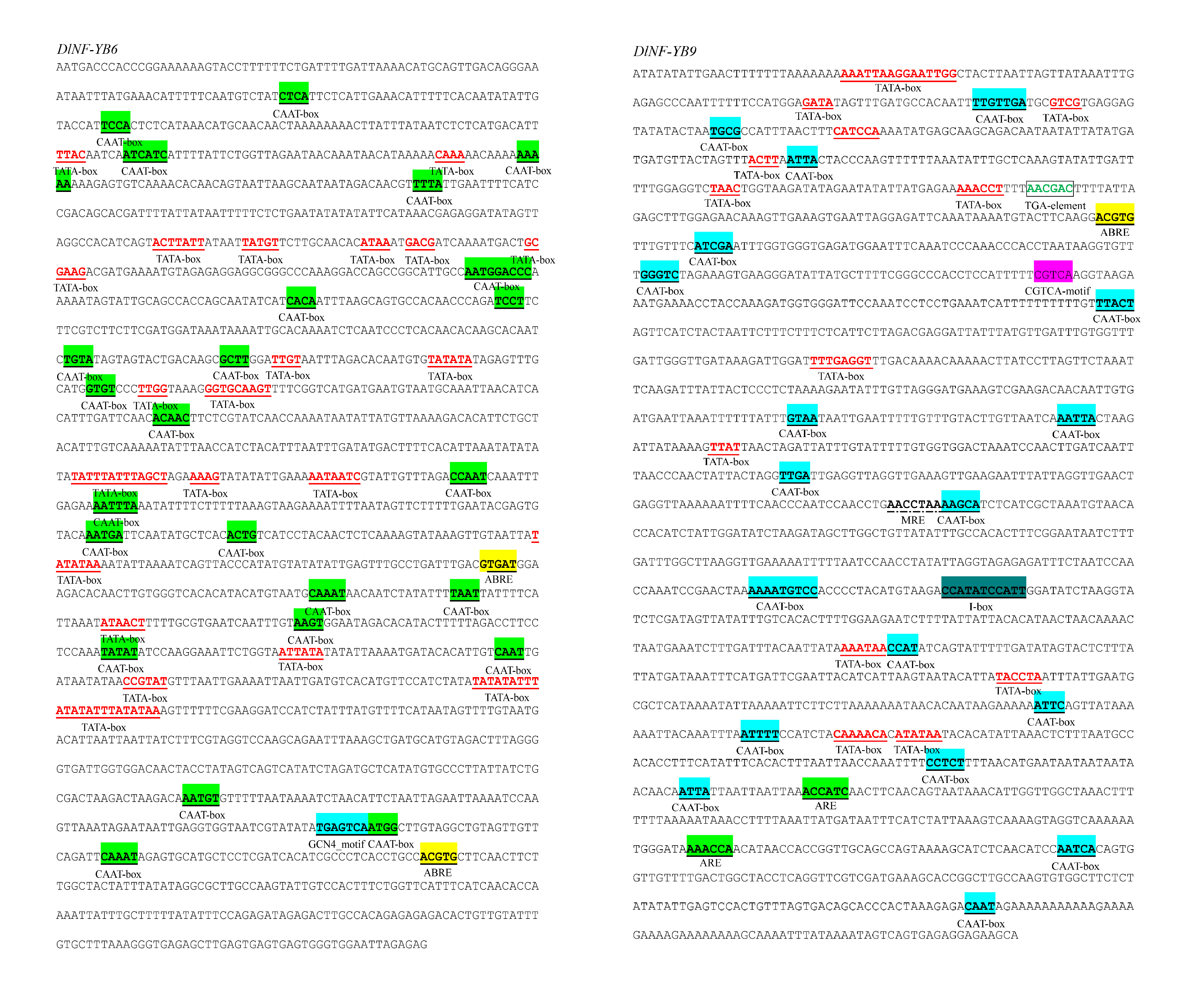

Supplement: Supplementary Figure 1 — The nucleotide sequence of DlNF-YB6 and DlNF-YB9 promoter. Note: The putative regulatory elements and hormone-responsive elements on both strands are shown in bold and underlined, which was performed by the PlantCARE database. [file Image_1.jpeg]
